# Diagnostic performance of shear wave elastography in thyroid nodules with indeterminate cytology: A systematic review and meta-analysis

**DOI:** 10.1016/j.heliyon.2023.e20654

**Published:** 2023-10-05

**Authors:** Yuxuan Qiu, Zhichao Xing, Qianru Yang, Yan Luo, Buyun Ma

**Affiliations:** a Department of Ultrasound, West China Hospital, Sichuan University, Chengdu, China; b Department of Thyroid & Parathyroid Surgery, West China Hospital, Sichuan University, Chengdu, China

**Keywords:** Thyroid nodule, Elasticity imaging techniques, Cytological techniques, Meta-analysis

## Abstract

**Purpose:**

Thyroid nodules classified as indeterminate in previous fine-needle aspiration cytology often necessitate additional evaluation to determine their histology, while shear wave elastography (SWE) offers an alternative option in this regard. The objective of this study was to assess the diagnostic effectiveness of SWE in evaluating indeterminate nodules.

**Methods:**

The PubMed, EMBASE, and Web of Science databases were searched from 1st January 1970 to 1st March 2023. The studies were reviewed and the data was extracted by two separate reviewers. A Bayesian bivariate model was utilized to quantitatively synthesize the diagnostic accuracy and yield of the studies in R.

**Results:**

A total of seven studies, involving indeterminate thyroid nodules undergoing SWE were included, and the overall malignancy rate was 34.1% (307/900). The summarized estimates of sensitivity and specificity were 0.792 (95% credible interval [CI], 0.727–0.850) and 0.845 (95% CI, 0.797–0.887), respectively. The summarized estimate for the diagnostic odds ratio (DOR) was 17.8 (95% CI, 14.0–22.6). Summarized receiver operating characteristic (SROC) plots indicated a trade-off between sensitivity and specificity, and the estimate of AUC was 0.866 (95% CI, 0.834–0.895). The summary estimates for positive and negative likelihood ratios were 4.67 (95% CI, 3.98–5.85) and 0.26 (95% CI, 0.23–0.28), respectively.

**Conclusions:**

The overall accuracy of SWE remains satisfactory in indeterminate thyroid nodules. However, it should be noted that the available data are still extremely limited, and more studies or guidelines are required to provide further insights.

## 1 Introduction

Thyroid nodules are a common occurrence in the adult population, with a prevalence of up to 60% and a cancer incidence of approximately 5% [1]. Ultrasound-guided fine needle aspiration cytology (FNAC) is the established diagnostic modality for preoperative evaluation of thyroid nodules [2]. However, it should be noted that this procedure may yield unsatisfactory and indeterminate results in up to 30% of cases [3,4]. Indeterminate categories commonly encompass a range of thyroid nodules, including atypia of undetermined significance and follicular lesion of uncertain significance (AUS/FLUS), follicular neoplasm or suspicious for a follicular neoplasm (FN/SFN), and suspicious for malignancy (SM) [5]. The management of thyroid nodules with indeterminate cytology poses ongoing difficulties in clinical practice. The key challenge lies in accurately identifying patients at a higher risk of cancer development, while minimizing unnecessary surgical interventions [6]. Ultrasonography (US), molecular studies, and core needle biopsy (CNB) are increasingly employed in conjunction with FNAC to enhance diagnostic accuracy and facilitate optimal therapeutic decision making [7]. Molecular testing can improve risk stratification, and there is currently a lack of long-term outcome data to fully assess its value in guiding therapeutic decision making [8]. CNB is increasingly being considered a viable alternative to FNAC, particularly in cases of AUS/FLUS and nondiagnostic cytology, such as calcified nodules [9]. However, CNB is a more invasive procedure than FNAC in conventional cognition and may not be a feasible option for managing small indeterminate nodules, thus limiting its applicability.

US elastography was utilized using stiffness as an indicator of malignancy for elastography, based on the observation that a suspicious nodule is palpably firm or hard in consistency [10,11]. Shear wave elastography (SWE) provides real-time measurement of tissue elasticity, quantified as an elasticity index (EI), along with a qualitative color-coded elasticity map [12]. Compared to other elasticity methods, SWE is less user-dependent because SWE utilizes acoustic pulses generated by a transducer to assess elasticity rather than relying on external pressure applied by the examiner [12,13]. Many studies have demonstrated that SWE exhibits superior performance in predicting the malignancy of thyroid nodule [14,15], and is also considered particularly valuable for evaluating indeterminate nodules or nodules with a follicular growth pattern [16,17]. Nonetheless, the usefulness of SWE in distinguishing thyroid nodules with indeterminate cytology remains a topic of controversy. Bardet et al. reported a sensitivity of 0.85 and a specificity of 0.94 with a cut-off value of maximum EI at 65 kPa using SWE in 131 indeterminate nodules [18]. Samir et al. reported a sensitivity of 0.85 and specificity of 0.94 in 35 indeterminate nodules but used a lower mean EI at a 22.3 kPa cutoff [19].

Therefore, we conducted this systematic review and meta-analysis to explore and summarize the diagnostic efficacy of SWE in thyroid nodules with indeterminate cytology along with the focus on its clinical applicability.

## 2 Methods

This systematic review and meta-analysis adhered to the Preferred Reporting Items for Systematic Review and Meta-Analyses (PRISMA) extension for diagnostic test accuracy statement [20].

### 2.1 Literature search

We conducted a comprehensive search across multiple databases, including PubMed, EMBASE, and Web of Science, covering the period from 1st January 1970 to 1st March 2023. We utilized “intermediate”, “indeterminate”, “undetermined”, “shear wave elastography”, “thyroid” and their synonyms as search terms. Additionally, we also examined the references of eligible studies and review articles to ensure comprehensive coverage of relevant literature.

### 2.2 Inclusion and exclusion criteria

First, studies or their subsets that examined the use of SWE to predict malignant thyroid nodules in patients who had previously received an indeterminate FNAC report were eligible for inclusion. Then, indeterminate nodules that underwent final postoperative histopathologic examination or repeated diagnostic FNAC were included. The exclusion criteria were as follows: a) articles that did not pertain to the field of interest (articles that did not include indeterminate nodules; articles that did not utilize SWE; indeterminate nodules were not validated by the final gold standard); b) reviews, case reports, editorials or letters, comments, and conference proceedings; and c) articles that were not written in English.

## 3 Data extraction

One investigator was responsible for extracting the descriptive data, which were then cross-checked and confirmed by another investigator. The extracted descriptive data included the encompassed information of the study, the details of the patients involved, and test characteristics. Two reviewers independently extracted the numerical data. Any discrepancies were resolved through discussion and consensus. In cases where the data were not readily extractable, we reached out to the authors for additional data that might be needed.

### 3.1 Risk of bias

The risk of bias and concerns about applicability were assessed by two independent reviewers using the Quality Assessment of Diagnostic Accuracy Studies 2 (QUADAS-2) tool [21]. Discrepancies were resolved through consensus. The inclusion of each study was determined through a comprehensive discussion between the two reviewers.

### 3.2 Data synthesis

In our meta-analysis, we applied a Bayesian bivariate model of diagnostic test studies using integrated nested Laplace approximation (INLA) [22]. INLA is a powerful computational method that allows for efficient and accurate estimation of parameters in Bayesian models and directly provides accurate posterior marginal distributions for sensitivity and specificity, as well as all hyperparameters and covariates, with no need for conventional Markov chain Monte Carlo sampling [23,24]. Furthermore, univariate results of sensitivity and specificity accompanied by 95% credible intervals (CIs), as well as the summarized receiver operating characteristic (SROC) curve, were directly available. Additionally, area under the receiver operating characteristic curve (AUC) values with 95% CI were combined. The summarized positive and negative likelihood ratios (LR + s and LR-s, respectively) were calculated from the summarized sensitivity and specificity estimates. The diagnostic odds ratios (OR) and risk difference (RD) were also derived from these estimates. Spearman correlation was utilized to test for the presence of a threshold effect, and a P value less than 0.05 was considered to be a significant threshold effect. Forest plots were utilized to visually depict the summarized sensitivity and specificity, along with the individual sensitivity and specificity of each study. Funnel plot asymmetry was also assessed to evaluate the degree and significance of publication and selective reporting bias. Subgroup analyses were performed according to the study design (prospective or retrospective), prevalence of malignancy (above or below the median rate), histopathology (surgery or repeated FNAC & surgery), different SWE systems (Young's moduli (Kpa) or shear wave velocity [SMV(m/s)]) and different EI (mean or maximum). All the analyses were conducted using R software 4.2.3 (R Foundation for Statistical Computing, Vienna, Austria; https://www.r-project.org) along with the R package meta4diag (2.1.1) and INLA (22.12.16), and their relied packages.

## 4 Results

### 4.1 Literature search and study characteristics

**Fig. 1:**
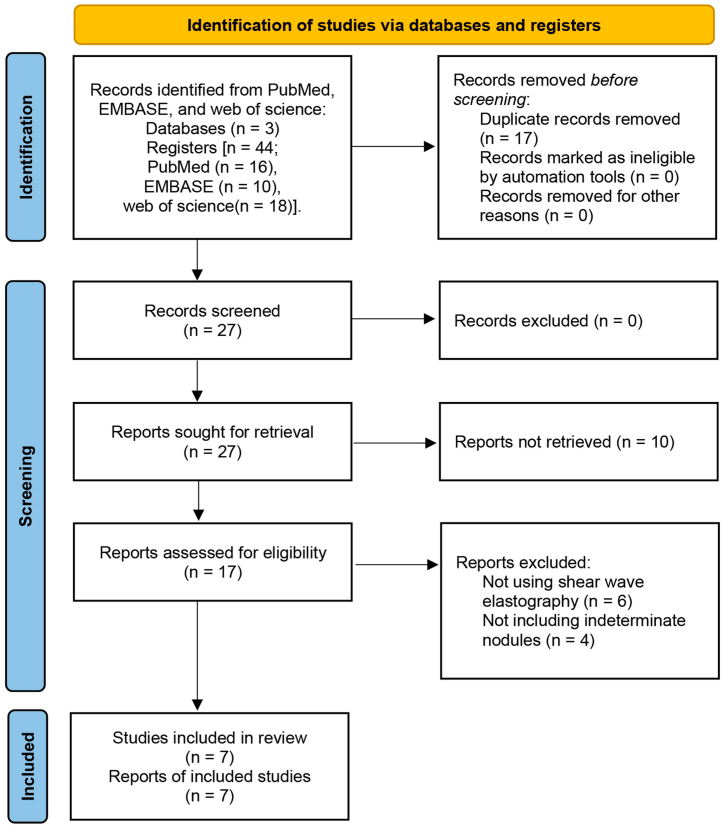
PRISMA diagram of study selection.

After removing duplicate results and conducting abstract screening, the potentially eligible full publications were thoroughly reviewed. After exclusions, a total of 7 studies were finally included in this systematic review [16,18,19,[25], [26], [27], [28]]. The study selection process is described in Fig. 1. Of the 7 studies, 5 were prospective, and 2 were retrospective. Among the 919 included patients, 932 thyroid nodules were initially classified as indeterminate by FNAC, and 900 of them were diagnosed by postoperative histopathology (5 articles) or both repeat FNAC and postoperative histopathology (2 articles). Of the 900 indeterminate nodules that were included in the final statistical analysis, 307 (34.1%) were found to be malignant. The prevalence of malignancy varied across the included studies, ranging from 14.2% to 80.8%, with a median prevalence of 29.2%. All details of the included studies are summarized in Table 1. Detailed information regarding SWE used and the diagnostic four-fold data of the included studies are provided in Table 2.

**Table 1:** Study characteristics.

Study (author, year)	Country	Study Design	Patients Enrolled Period	No. Patients	No. Female (%)	Age (SD/Range)	No. of Indeterminate Nodules in Final Diagnosis	Diameter of Indeterminate Nodules	FNAC Classification	Final Diagnosis	Malignant rate (%)
Samir et al., 2015 [19]	USA	Prospective	2010.4–2012.4	35	23 (65.7)	55 (16.1)	38	≥10 mm	Bethesda (III, IV)	Repeated FNAC or Surgery	31.4%
Bardet et al., 2017 [18]	France	Prospective	2013.5–2015.9	131	98 (74.8)	52 (15)	131	≥15 mm	Bethesda (III–V)	Surgery	16.0%
Azizi et al., 2018 [27]	USA	Prospective	2014.4–2016.10	151	135 (89.4)	51.4 (15.77)	151	≥5 mm	Bethesda (III, IV)	Repeated FNAC or Surgery	20.5%
Chen et al., 2019 [28]	China	Retrospective	2014.3–2017.8	223	187 (83.9)	54 (18.4)	232	≥5 mm	Bethesda (I, III)	Surgery	14.2%
Zhang et al., 2020 [16]	China	Retrospective	2014.1–2019.12	193	152 (78.8)	46.1 (13.1)	193	≥5 mm	Bethesda (III–V)	Surgery	80.8%
Celletti et al., 2021 [26]	Italy	Prospective	2017.1–2018.2	128^a^	89 (69.5)	54.3 (18–82)	96	Any	TIR (3A, 3B)	Surgery	29.2%
Moraes et al., 2021 [25]	Brazil	Prospective	2016.12–2018.7	58	51 (87.9)	54.7 (14)	62	Any	Bethesda (III, IV)	Surgery	43.5%

a The study solely presents the initial number of included patients and does not provide the final count; FNAC, fine-needle aspiration cytology; USA, United States of America.

**Table 2:** Details for SWE used in the included studies.

Study (author, year)	SWE system	Optimal Parameters	Cut-off value	TP	FP	FN	TN	Sensitivity	Specificity	PPV	NPV
Samir et al., 2015 [19]	Supersonic Imagine Aixplorer	Emean	22.3 KPa	9	3	2	24	0.818	0.889	0.750	0.923
Bardet et al., 2017 [18]	Supersonic Imagine Aixplorer	Emax	65 KPa	18	7	3	103	0.857	0.936	0.720	0.972
Azizi et al., 2018 [27]	Siemens Acuson S300	Mean SWV	3.59 m/s (38.7Kpa)^a^	26	25	5	95	0.839	0.792	0.510	0.950
Chen et al., 2019 [28]	Toshiba Aplio 500	Emean	24 KPa	26	30	7	169	0.788	0.849	0.464	0.960
Zhang et al., 2020 [16]	Supersonic Imagine Aixplorer	Emax	41.2 KPa	126	8	30	29	0.808	0.784	0.940	0.492
Celletti et al., 2021 [26]	Toshiba Aplio 500/800	Emean	36.8 KPa	16	14	12	54	0.571	0.794	0.533	0.818
Moraes et al., 2021 [25]	GE Logiq	Mean SWV	3.62 m/s (39.2 KPa)^a^	22	6	5	29	0.815	0.829	0.786	0.853

where c is shear wave speed in m/s and p is tissue density (a constant = 1000 kg/m3).

SWE, shear wave elastography; SWV, shear wave velocity; Emean, mean elastic index; Emax, maximum elastic index; TP, true positive; FP, false positive; FN, false negative; TN, true positive; PPV, Positive Predictive Value; NPV, Negative Predictive Value.

a SWV measurement can be converted from m/s to kilopascals (kPa) using the following formula: kPa (Young's modulus) = 3pc^2^.

### 4.2 Risk of bias

**Fig. 2:**
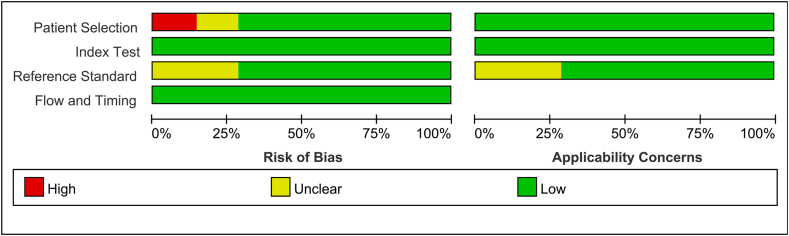
Quality assessment of the included studies according to the Quality Assessment of Diagnostic Accuracy Studies-2 (QUADAS-2) criteria.

Two reviewers conducted an independent assessment of the risk of bias and concerns about applicability based on the QUADAS-2 tool. The results of the quality assessment are presented in Fig. 2. One article only enrolled indeterminate nodules over 15 mm in diameter, and had a high risk of patient selection [18]. Overall, the applicability of the included studies was fine for inclusion.

### 4.3 Diagnostic performance

**Fig. 3:**
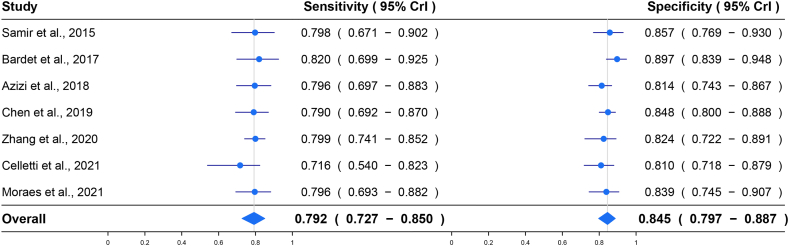
Forest plots of the summarized estimates of sensitivity and specificity.

**Fig. 4:**
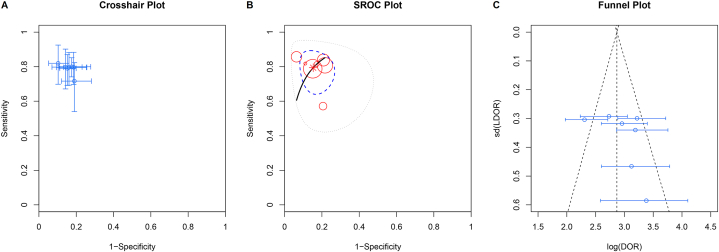
A) Crosshair plots show reported prior-point estimates (shown as circles) and confidence intervals (shown as extended lines). B) Summary receiver operating characteristic (SROC) curve showing individual study posterior-point estimates (the size of each circle is proportional to the sample size for each study). The dashed elliptical boundary represents the 95% credible region for the summary estimates (closed diamond). The standard (black) and latent class model analyses based on the conditional dependence model (blue) and the conditional independence model (red) are presented. C) Funnel plot for bias. (For interpretation of the references to color in this figure legend, the reader is referred to the Web version of this article.)

The summarized results for sensitivity and specificity were 0.792 (95% CI, 0.727–0.850) and 0.845 (95% CI, 0.797–0.887), respectively (Fig. 3). Crosshair plot indicated the sensitivity and specificity across different studies are consistent (Fig. 4A). The summary results for LR+ and LR-were 4.67 (95% CI, 3.98–5.85) and 0.26 (95% CI, 0.23–0.28), respectively. The summarized results for OR and RD were 17.8 (95% CI, 14.0–22.6) and 0.61 (95% CI, 0.58–0.64), respectively. The estimate of AUC was 0.866 (95% CI, 0.834–0.895), and the corresponding SROC plot is shown in Fig. 4B. In addition, no threshold effect was found by the Spearman correlation test (P = 0.383), and no possible publication bias was found according to the funnel plot, as shown in Fig. 4C. The results of subgroup analyses are displayed in Table 3. In terms of AUC, prospective studies, lower malignant rate, total surgical histopathology and SWE systems providing EI rather than SWV offered better diagnostic value.

**Table 3:** Subgroup analyses.

	AUC (95% CI)	Sensitivity (95% CI)	Specificity (95% CI)	LR+ (95% CI)	LR- (95% CI)	OR (95% CI)	RD (95% CI)
Study design							
Prospective	0.881 (0.866–0.915)	0.782 (0.677–0.873)	0.853 (0.782–0.911)	5.47 (4.92–6.20)	0.32 (0.20–0.40)	18.4 (12.3–31.1)	0.59 (0.52–0.69)
Retrospective	0.712 (0.559–0.865)	0.801 (0.695–0.881)	0.829 (0.720–0.902)	3.52 (3.26–3.77)	0.44 (0.23–0.64)	11.0 (5.3–16.7)	0.45 (0.31–0.60)
Prevalence of malignance							
<29.2%	0.889 (0.860–0.918)	0.769 (0.643–0.874)	0.848 (0.768–0.912)	5.65 (4.64–6.51)	0.24 (0.18–0.33)	25.8 (14.6–32.2)	0.65 (0.57–0.70)
>29.2%	0.855 (0.785–0.893)	0.811 (0.726–0.880)	0.831 (0.738–0.903)	4.94 (4.31–5.73)	0.25 (0.18–0.35)	22.1 (12.6–31.9)	0.63 (0.54–0.70)
Determinate by histopathology							
Surgery	0.894 (0.862–0.930)	0.781 (0.692–0.855)	0.852 (0.789–0.902)	6.16 (4.29–9.25)	0.22 (0.16–0.29)	31.2 (15.2–59.2)	0.67 (0.59–0.76)
Repeated FNAC & surgery	0.847 (0.846–0.848)	0.832 (0.677–0.931)	0.820 (0.701–0.920)	4.31 (3.85–4.78)	0.24 (0.17–0.31)	20.8 (12.5–29.1)	0.62 (0.55–0.68)
SWE systems							
Young's moduli (Kpa)	0.896 (0.884–0.908)	0.780 (0.685–0.859)	0.860 (0.798–0.910)	5.59 (4.21–6.19)	0.25 (0.20–0.31)	22.9 (20.1–27.7)	0.64 (0.61–0.68)
SMV (m/s)	0.823 (0.792–0.853)	0.827 (0.693–0.919)	0.802 (0.696–0.888)	3.47 (3.44–3.49)	0.29 (0.26–0.32)	12.1 (10.9–13.3)	0.55 (0.53–0.57)
Different EI							
Mean	0.843 (0.815–0.869)	0.767 (0.668–0.853)	0.826 (0.780–0.868)	4.19 (3.49–5.34)	0.30 (0.22–0.35)	14.8 (10.1–24.9)	0.57 (0.52–0.66)
Maximum	0.879 (0.843–0.914)	0.820 (0.713–0.907)	0.870 (0.617–0.973)	3.76 (2.62–4.89)	0.21 (0.16–0.26)	20.7 (10.4–31.1)	0.60 (0.51–0.69)
Overall	0.866 (0.834–0.895)	0.792 (0.727–0.850)	0.845 (0.797–0.887)	4.67 (3.98–5.85)	0.26 (0.23–0.28)	17.8 (14.0–22.6)	0.61 (0.58–0.64)

AUC, area under curve; LR+, positive likelihood ratio; LR-, negative likelihood; OR, odds ratio; RD, risk difference; SWE, shear wave elastography; EI, elastic index; SWV, shear wave velocity; FNAC, fine-needle aspiration cytology.

## 5 Discussion

The incidence of thyroid nodules with indeterminate cytology varies widely in the literature. Bethesda III and IV can account for up to approximately 50% of the first FNAC results (35% for AUS/FLUS and 22.1% for FN/SFS), while rates as low as 0.78% for AUS/FLUS and 8.1% for FN/SFN have also been reported [29,30]. The Bethesda system also recommends considering repeat FNA, molecular testing, or lobectomy for indeterminate nodules [31]. On the whole, the current management of intermediate nodules involves individual assessment based to the US risk category of the nodule and the presence of suggestive clinical and historical/anamnestic risk factors [8,32]. If repeat FNAC produces an indeterminate or nondiagnostic result, the ensuing follow-up is the first consideration, and lobectomy can also be an alternative if there is no absolute contraindication to surgery. Therefore, if an additional noninvasive tool could effectively distinguish benign nodules from indeterminate nodules, unnecessary invasive diagnostic procedures or lobectomy could be avoided.

In recent years, SWE has been proposed to identify benign and malignant nodules by quantitative elasticity values, which can display the difference in hardness intuitively. Several studies have demonstrated a correlation between lower elasticity of thyroid nodules and a higher likelihood of malignancy. However, it is important to note that this correlation could potentially be attributed to other histological features as well [33,34]. Many studies and an updated meta-analysis have evaluated the diagnostic value of SWE and showed that SWE is a useful tool for identifying thyroid nodules [11]. In this particular context, a few papers have examined the reliability and effectiveness of SWE in patients with indeterminate thyroid nodules. In this meta-analysis, 7 studies were included, and we yielded promising findings suggesting that SWE was an optimal tool for the diagnostic evaluation of thyroid nodules with indeterminate cytology. The summarized results for sensitivity and specificity were 0.792 (95% CI, 0.727–0.850) and 0.845 (95% CI, 0.797–0.887), respectively. In the subgroup analysis, the results did not suggest significantly different sensitivity or specificity in any given subgroup, which might mean that SWE has better universality in all used scenarios. However, these findings were not as expected in comparison with our previous study, where SWE showed a sensitivity of 0.838 and a specificity of 0.872 in distinguishing indeterminate nodules [35]. The previous study only included 3 articles of SWE, and sample sizes were not sufficient (317 nodules); thus, we thought it was no more consistent than our new findings.

Although the diagnostic efficiency of SWE has been demonstrated in this paper, a significant controversy currently lies in the choice of the EI and the determination of the cut-off value. We noticed that 5 included studies reported the optimal diagnostic value of mean EI, while 2 chose maximum EI. We suspect this might be due to the fact that indeterminate cytology is often associated with follicular carcinoma, and this could potentially result in the preselection of less stiff lesions, leading to more accurate diagnostic outcomes in the included studies. Therefore, mean EI could be more suitable for indeterminate cytology. On the other hand, studies that specifically excluded patients with indeterminate cytology showed a higher stiffness of malignant lesions. This could be attributed to a higher percentage of papillary thyroid carcinoma, where the maximum EI might be a more informative parameter [36,37]. This could also explain what we found in the subgroup analysis where maximum EI seemed to be more reliable because Zhang et al. reported very high malignancy (80.8%) and more papillary cancers compared to other included articles [16]. In addition, the cut-off values were highly variable in our included articles. At present, the dispute cannot be resolved because there are many factors that affect the cut-off value. Studies that included a substantial number of cases with papillary thyroid carcinoma often reported the highest cut-off points for EI [36,38]. Nodule size is another factor where a higher EI was found in larger nodules [37,39]. In addition, It has been noted that the presence of microcalcifications can influence the interpretation of SWE [40]. Therefore, further study is warranted to standardize the cut-off values for wider clinical use and provide guidance in interpreting thyroid nodule results.

The updated EFSUMB guidelines recommended using US elastography for characterizing thyroid nodules due to its high positive predictive value and for follow-up of patients with cytologically highly suggestive benign nodules [41]. However, the use of US elastography in evaluating indeterminate thyroid nodules is not currently recommended due to limited available data. In this paper, the role of SWE was further confirmed in indeterminate thyroid nodules. However, we also noticed some conflicts in comparison with other US elastography techniques. Celletti et al. reported both higher sensitivity (0.857 vs 0.571) and specificity (0.941 vs 0.794) in strain ratio elastography than SWE [26]. Gay et al. conducted a study on the multiparametric assessment of indeterminate thyroid nodules and found no significant correlation between strain ratio elastography and SWE [42]. Our previous study found comparable values for both strain ratio elastography and SWE [35]. Thus, we thought different US elastography techniques are not strictly surrogate relationships, and it will make more sense to refer selectively to the results of one single device or to combine them.

Some limitations should be briefly discussed here. Despite the adoption of a Bayesian method to minimize heterogeneity, the limited number of included studies might still amplify heterogeneity. The malignancy rate was 34.1% in our study, which was higher than that expected in indeterminate thyroid nodules. Agreement between repeated measurements is not discussed in this study, which could influence the performance, although SWE is often considered to be user independent.

## 6 Conclusion

In conclusion, the overall accuracy of SWE remains satisfactory in indeterminate thyroid nodules. However, the available data are still extremely limited, and further studies are needed. We hope that future guidelines can reach some consensus regarding the role of SWE in indeterminate thyroid nodules.

## Data availability statement

Data included in article/supp. material/referenced in article.

## Funding

This study received no funding support.

## CRediT authorship contribution statement

**Yuxuan Qiu:** Data curation, Formal analysis, Investigation, Methodology, Project administration, Resources, Software, Supervision, Validation, Visualization, Writing – original draft, Writing – review & editing. **Zhichao Xing:** Conceptualization, Formal analysis, Investigation, Resources, Writing – original draft, Writing – review & editing. **Qianru Yang:** Formal analysis, Project administration, Writing – original draft, Writing – review & editing. **Yan Luo:** Conceptualization, Writing – original draft, Writing – review & editing. **Buyun Ma:** Conceptualization, Writing – original draft, Writing – review & editing.

## Declaration of AI and AI-assisted technologies in the writing process

During the preparation of this work no authors used AI or AI-assisted technologies.

## Declaration of competing interest

The authors declare that they have no known competing financial interests or personal relationships that could have appeared to influence the work reported in this paper.
